# Characteristics of step responses following varying magnitudes of unexpected lateral perturbations during standing among older people – a cross-sectional laboratory-based study

**DOI:** 10.1186/s12877-022-03080-w

**Published:** 2022-05-06

**Authors:** Shani Batcir, Guy Shani, Amir Shapiro, Itshak Melzer

**Affiliations:** 1 Department of Physical Therapy, Faculty of Health Sciences, Ben-Gurion University of the Negev, Beer-Sheva, Israel; 2 Department of Information Systems, Faculty of Engineering Sciences, Ben-Gurion University, Beer-Sheva, Israel; 3 Department of Mechanical Engineering, Faculty of Engineering, Ben-Gurion University of the Negev, Beer-Sheva, Israel

**Keywords:** Falls, Older adults, Unexpected balance perturbation, Step recovery response, Total balance recovery, First step is completed

## Abstract

**Introduction:**

The inability to recover from unexpected lateral loss of balance may be particularly relevant to the problem of falling.

**Aim:**

We aimed to explore whether different kinematic patterns and strategies occur in the first recovery step in single-step trials in which a single step was required to recover from a fall, and in multiple-step trials in which more than one step was required to recover from a fall. In addition, in the multiple-step trials, we examined kinematic patterns of balance recovery where extra steps were needed to recover balance.

**Methods:**

Eighty-four older adults (79.3 ± 5.2 years) were exposed to unannounced right/left perturbations in standing that were gradually increased to trigger a recovery stepping response. We performed a kinematic analysis of the first recovery step of all single-step and multiple-step trials for each participant and of total balance recovery in the multiple-step trial.

**Results:**

Kinematic patterns and strategies of the first recovery step in the single-step trials were significantly dependent on the perturbation magnitude. It took a small, yet significantly longer time to initiate a recovery step and a significantly longer time to complete the recovery step as the magnitude increased. However, the first recovery step in the multiple-step trials showed no significant differences between different perturbation magnitudes; while, in total balance recovery of these trials, we observed a small, yet significant difference as the magnitude increased.

**Conclusions:**

At relatively low perturbation magnitudes, i.e., single-step trials, older adults selected different first stepping strategies and kinematics as perturbation magnitudes increased, suggesting that this population activated pre-planned programs based on the perturbation magnitude. However, in the first recovery step of the multiple-step trials, i.e., high perturbation magnitudes, similar kinematic movement patterns were used at different magnitudes, suggesting a more rigid, automatic behavior, while the extra-steps were scaled to the perturbation magnitude. This suggest that older adults activate pre-planned programs based on the magnitude of the perturbation, even before the first step is completed..

**Supplementary Information:**

The online version contains supplementary material available at 10.1186/s12877-022-03080-w.

## Introduction

A sideways fall caused by an unexpected lateral loss of balance is more likely to result in direct ground contact with the greater trochanter, possibly resulting in hip fracture [1–3]. Previous studies show more prominent impairments in the balance control system in the frontal plane, i.e., ML-direction [4, 5], and that measuring lateral instability provides better predictors of falls and even injurious falls [6, 7]. The importance of measuring balance in the frontal plane is further increased since many falls involve lateral falls [8, 9], resulting in hip fractures [8]. Avoiding lateral falls is equally essential compared with BMI [10–12]. Most research on mediolateral balance reactive control has focused mainly on the first recovery step in mediolateral perturbation, and at a specific perturbation magnitude [2, 13–16]. Several research studies have been conducted of older participants exposed to increasing magnitudes of mediolateral perturbations [2, 17–25]. In these investigations, it was found that in cases in which the perturbation magnitudes were low, a fixed base of support strategies were used to preserve balance. At higher perturbation magnitudes, a change in base-of-support strategies were used, i.e., a single-recovery step response; and in higher perturbation magnitudes extra steps were needed to preserve balance, i.e., a multiple-step response. Recently, Fujimoto et al. [21] investigated if balance stability at first-step initiation (i.e., first step lift-off) differed between multiple- and single-step responses to lateral perturbations in older adults who received lateral waist-pulls at five different magnitudes of perturbations. They found that compared to younger people, older adults had reduced stability at the first foot contact that was associated with taking additional steps. More recently, Batcir et al. [22] found that older adults who reported several falls showed a significant delay in step initiation duration, and had longer step duration and a larger center of mass (CoM) displacement during single-step trials compared with non-fallers and one-time fallers. In their multiple-step threshold trials, when extra steps were needed to recover balance, the participants who had reported several falls exhibited larger CoM displacements and took a longer time to fully recover from balance loss [22]. It is thought that these stepping responses could also be attributed to the CoM motion state as early as the first step lift-off, preceding foot contact [22]. The kinematic characteristics of the first recovery step (e.g., step timing, length, velocity, and acceleration) need to match the requirements for optimal control of stability in different conditions, i.e., perturbation magnitudes [26].

In the present study, we sought to investigate the kinematic patterns of the recovery step in single-stepping responses and of the first recovery step in multiple-stepping responses when right/left perturbations systematically increased from very low magnitudes to very high magnitudes in standing. This would provide a clearer understanding of the dynamics of recovery step responses in older people, and can deepen insight into the underlying balance control mechanisms of balance recovery. Earlier, Vlutters et al. [25] exposed young people to right/left and forward/backward pelvic perturbations at various magnitudes at two different treadmill walking speeds (i.e., low and high). They found that foot placement after right/left perturbations was adjusted proportionally to the right/left CoM velocity, whereas forward/backward unexpected perturbations did not show a similar response. Nachmani et al. [27] found that as the perturbation magnitudes increased during self-selected treadmill walking, older adults showed a small, yet significant decreases in the timing of the step response, and increased their step length

To truly demonstrate the existence of different control patterns at different perturbation magnitudes, in our experimental set-up, we adjusted the magnitude of perturbations from very low to very high, and set different onset timings and directions of the perturbations, i.e., right/left. We hypothesized that older people would show similar timing for their first recovery step initiation and step duration at different perturbation magnitudes, suggesting that the temporal patterns of the recovery step response are stereotypic and almost automatic in nature. We also hypothesized that spatial parameters of the first recovery step such as step length, step velocity, and CoM displacement would be adjusted proportionally to the magnitude of the right/left perturbation. In regard to multiple-step trials, i.e., higher perturbation magnitudes, we hypothesized that the total time to recover balance would be scaled to the magnitude of right/left perturbations and be more adaptive.

## Methods

### Participants

Eighty-four older adults were recruited for two randomized control trials that were approved by the Helsinki Committee of Barzillai University Medical Center in Ashkelon, Israel (ClinicalTrials.gov registration number #NCT01439451, initial release 23/09/2011 and ClinicalTrials.gov registration number #NCT03636672 initial release 17/08/2018). The analysis was a supplementary study based on the baseline measures of the two randomized control trials. Participants were independent older adults aged 70 years old and over. The exclusion criteria were: hip or knee arthroplasty within the prior year, Mini Mental State Examination score (MMSE) < 24 [28], visual blindness, vestibular dizziness, severe peripheral neuropathies, severe arthritis in the lower limbs, symptomatic orthostatic hypotension, respiratory diseases, stroke, Parkinson’s disease, multiple sclerosis, amyotrophic lateral sclerosis, or cancer under active treatment.

### Study protocol

The participants signed the consent form, then they stood with their heels and toes touching on a i.e., a motor-driven perturbation treadmill device (i.e., Balance Measure & Perturbation System) that in the presnt experiment provided right or left unannounced surface translation in standing condition [29]. They were exposed to a total of 26 random right and left unannounced surface translation perturbations that systematically increased from low to high magnitudes (13 perturbation magnitudes). The specific instructions given to the subjects were "unconstrained" by any specific instructions i.e., react naturally. The magnitudes of 13 perturbations were specified in terms of transverse motion in cm of the Balance Measure & Perturbation System, thetiming in ms, the velocity in cm/ms, and the acceleration in cm/ms2 (details in supplementary Table 1). The order of unannounced perturbation times across the experimrnt and the direction were randomized, while the magnitudes were not. We performed this experimental setup since exposing old adults to high perturbation magnitude at an early stage of the experiment induced a stepping response at all perturbation magnitudes, which impairs our ability to identify step thresholds. The participants were instructed to stop the experiment or rest at any point. During the experiment, the participant wore a safety harness that did not influence lower extremity kinematics and prevented falling on the ground.

### Data analysis

The presence of single-step and multiple-step responses and the strategies of the first recovery step following a unannouced perturbations were verified offline using Windows Media Player (30 frames per-seconds), which allows video clip pauses, running the clip forwards and backwards, and slow motion. The following classifications were used [22, 26, 27]: Loaded-leg sideway stepping (LLSS)—the participant performs his/her initial step sideway with the loaded leg after the perturbation; Unloaded-leg sideway stepping (ULSS)–, the participant performshis/her initial step sideway with unloaded leg after the perturbation; Cross-over stepping (COS) the participant performed his/her stepping with the unloaded leg, while crossing the one leg over the other leg; Hip Abduction—the participant, abducted his/her hip joint of the unloaded leglateraly; Leg Collisions (Col). The unsuccessful balance recovery was defined as a fall into the safety harness system and grasping the harness system anchor straps or the research assistant to maintain their balance. The leg abduction and unsuccessful balance recovery reactions (i.e., fall events) were not analyzed.

3D kinematic data were captured in the single- and multiple-step trials with the Ariel Performance Analysis System (APAS, Ariel Dynamics Inc.; CA, USA) using two video cameras that simultaneously recorded the motion of 8 reflective markers that were placed at the anterior midpoint of the ankle joints, anterior superior iliac spines, acromion processes, and radial styloid processes (see a detailed description in [22, 26]). This approach was previously shown to be valid and reliable [30].

The kinematic parameters that were calculated: Step initiation duration (in milliseconds, ms) defined asthe time from perturbation to foot-off the ground initiating the recovery step; the first recovery step duration (ms), defined as the time from perturbation to contacting the ground with the foot completing the recovery step; and the length of the first recovery step defined as the distance that the foot marker moved in cm during the first recovery step. In case several steps were needed to recover balance, we calculated: The total balance recovery duration (ms), defines as the time from perturbation until the participant completed his/her balance recovery, performing several steps; Balnce recovery steps path-length (cm), defined as the distance that the foot marker moved in cm performing several steps to complete full balance recovery; and eCoM total path (cm), the estimated CoM dispalcment in cm to complete balance recovery when several steps were needed. The reliability of the emanination procedure is presented in detlais in Batcir et al. [26].

Age, MMSE [19], height, weight, BMI, number of medications taken per day, number of diagnosed diseases, gender proportion, number of falls in the last year, the Fall Efficacy Scale (FES-I) [31], late life function, and disability [32] were also assessed.

### Statistical analysis

All data were analyzed with PASW Statistics, version 26.0 (Somers, NY, USA). To examine our hypotheses, we tested the associations between the dependent spatial and temporal parameters of the first stepping response in single-step and multiple-step reactions separately (e.g., step initiation duration, step duration, step length, and step velocity) and the perturbation magnitudes using curve estimation linear regression. Since the step strategies may affect the kinematics of stepping, we adjusted the linear regression models for the first step strategy (ULLS, LLSS, and COS). In addition, we tested the associations between the dependent kinematic variables of the total balance recovery parameters during the multiple-step trials (e.g., total recovery duration, recovery step path length, and total eCoM displacement) and the perturbation magnitudes using curve estimation linear regression, adjusting the models for the first step strategy (ULLS, LLSS, and COS). Statistical significance was set a-priori at *p* < 0.05). The guidelines used to interpret correlation magnitudes were based on Cohen’s d = 0.15, 0.40, and 0.75 to interpret small, medium, and large effects [33].In order to better understand the mechanisms of balance recovery at increasing magnitudes of perturbation, we used a mosaic plot to explore the first step recovery strategies that were performed in the balance reactions. A mosaic plot is a graphical display of the cell frequencies of a contingency table in which the area of boxes of the plot are proportional to the cell frequencies of the contingency table. The widths of the boxes were proportional to the percentage of step responses performed out of the total stepping reactions. The heights of the boxes were proportional to the percent of the first step strategies used to recover from balance loss at each perturbation level. Comparisons of frequencies of step recovery strategies between single- and multiple-step reactions were made using Chi-square tests.

## Results

The participants' characteristics were: mean age 79.3 ± 5.2 years, 70% female (*n* = 59), mean MMSE 28.6 ± 1.4, number of medications a day 3.9 ± 2.1 (range 0–11), number of diagnosed diseases 1.8 ± 1.5, height 159.4 ± 9.7 cm, weight 67.8 ± 13.2 kg, BMI 26.7 ± 4.0 kg/m^2^, 38% (*n* = 32) reported a fall in the past year (range 0–4 fall events), FES-I 22.0 ± 7.9, and overall function score in the Late Life Function and Disability Instrument 66.1 ± 8.8. The single-step threshold was 8.2 ± 3.3, and the multiple-step threshold was 11.1 ± 3.6.

A total of 798 stepping trials were observed. We excluded 104 trials where the participants performed hip abduction responses and 17 trials where the participants fell into the harness system (unsuccessful balance recovery reactions). Thus, 447 single-step trials and 220 multiple-step trials were included in our analysis. Out of the 84 subjects, 55 completed the protocol (26 perturbation trials), 18 asked to stop before completing the experiment (they performed 20.6 ± 3.9 perturbation trials on average), and with 11 subjects, we encountered technical problems at the end of the study protocol (they performed 22.0 ± 2.3 perturbation trials on average that were successfully analyzed).

### Kinematic patterns of step recovery response

**Fig. 1:**
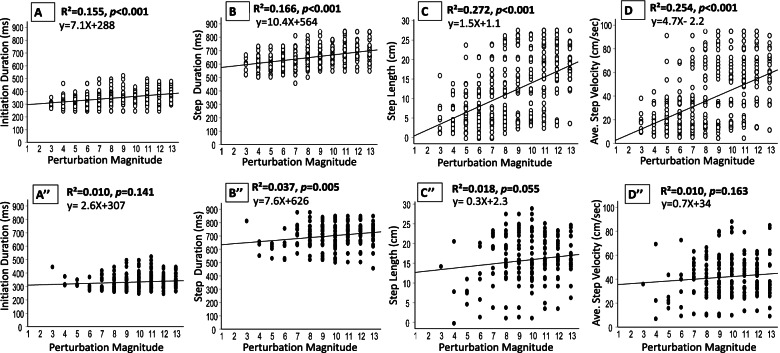
Kinematics of the first recovery step of single-step and multiple-step reactions at increasing perturbation magnitudes. Effect of increasing magnitude on the first recovery step parameters A–D show results for single-step reactions (white circles), A'–D' show results for multiple-step reactions (black circles). (A) and (A') represent step initiation durations, (B) and (B') represent step durations, and (C) and (C') are step length. Abbreviations: cm = centimeters; ms = milliseconds; sec = seconds

Figure 1A and B show that in single-step reaction trials, as the perturbation magnitudes were systematically increased, there was a small, yet significant increase in step initiation and step duration (R^2^ = 0.155, *p* < 0.001 and R^2^ = 0.166, *p *< 0.001, respectively). Additionally, the spatial parameters of recovery stepping, i.e., the step length and average step velocity, showed a larger increase (Fig. 1C, R^2^ = 0.272, *p* < 0.001 and Fig. 1D, R^2^ = 0.254, *p* < 0.001, respectively).

**Fig. 2:**
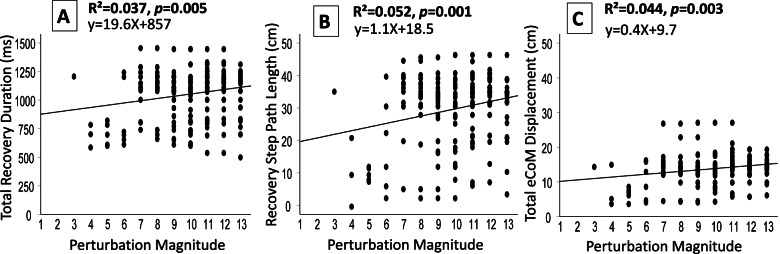
Kinematics of the total recovery step parameters at increasing perturbation magnitudes. Effect of increasing magnitude on the total recovery step parameters A–C show results for multiple-step reactions (black circles): (A) Total recovery step durations, (B) Total recovery step path length, and (C) Total estimated CoM displacement. Abbreviations: cm = centimeters; ms = milliseconds; sec = seconds

In regard to the first recovery step in the multiple-step trials, older adults did not show significant differences in step initiation, step length, or step velocity as the perturbation magnitudes were systematically increased (Figs. 1A' C' and D', but there was,a small, yet significant incease for step duration (R^2^ = 0.037, *p* = 0.005, Fig. B').

Figure 2A shows that when multiple steps were needed to recover balance, there was a small, yet significant increase in the total recovery step duration as the perturbation magnitudes increased (R^2^ = 0.037, *p* = 0.005). Also, the spatial parameters of the total balance recovery, i.e., the recovery step path length and the total eCoM displacement, showed a small, yet significant increase as the perturbation magnitude increased (Fig. 2 B and C, R^2^ = 0.052, *p* = 0.001 and R^2^ = 0.044, *p* = 0.003, respectively).

### The first recovery step strategies in single- and multiple-stepping trials

**Fig. 3:**
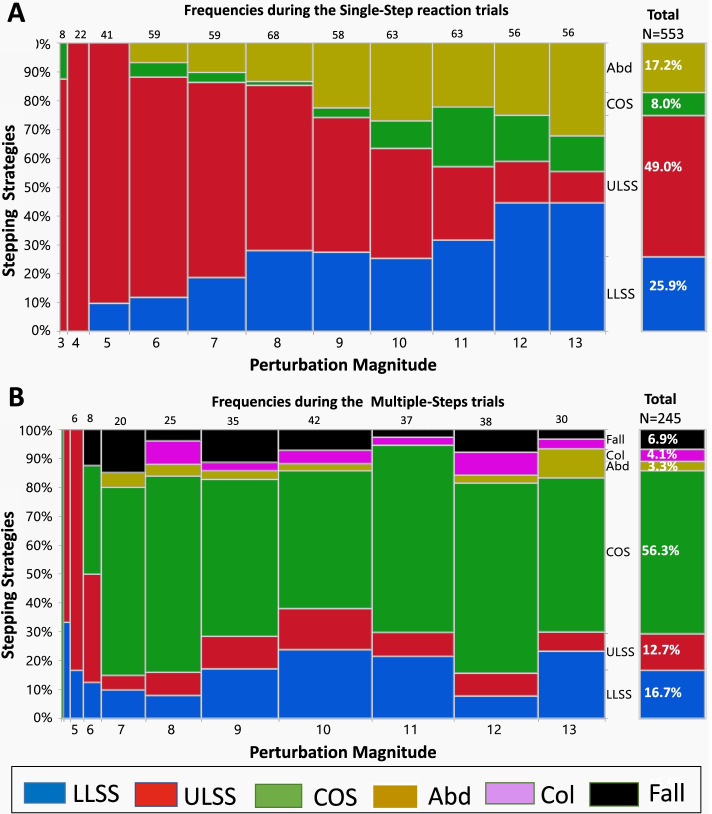
Mosaic plot of the first step strategies in single-step (A) and multiple-step (B) reactions. The mosaic plot is a graphical display of the leg strategy frequencies (Y-axis) by perturbation magnitudes (X-axis) during the two types of reactions. The widths of the boxes are proportional to the percentage of steps performed out of the total stepping reactions [the total number of single-step (*N*=553) and multiple-step reactions (*N*=245)] at each magnitude presented at the top of each graph. The heights of the boxes are proportional to the percent of the strategies used to recover from balance loss at each perturbation magnitude level. The isolated right columns summarize all the frequencies of the leg strategies at all magnitudes. Abbreviations: ULSS = unloaded leg side step; COS = cross-over step; Leg Abduction = abducting; a leg Col = leg collisions

Mosaic plots in Fig. 3 show the frequencies of the strategies performed in the first recovery step in the single-step and multiple-step trials (Fig. 3A, B). In the single-step trials, there was a gradual increase in the incidence of LLSS (blue boxes) and leg abduction (yellow boxes) strategies and a concurrent decrease in the ULSS strategy (red boxes). However, when multiple-steps were needed to recover balance (Fig. 3B), the strategy of the first recovery step appeared to be the same along all perturbation magnitudes.

In addition, when we compared the total frequencies of the first recovery step strategies between single- and multiple-step trials (Fig. 3A vs. Figure B, right isolated columns), a significant change in the response patterns was found. While the LLSS, ULSS, and leg abduction strategies were the most dominant in single-step reaction trials, these balance strategies were rarely performed during multiple-step reactions (25.9% vs. 16.7%, *p* = 0.004; 49% vs. 12.7%, *p* < 0.001; and 17.2% vs. 3.3%, *p* < 0.001, respectively). The COS strategy was rarely performed in the single-step reaction trials, but was the main strategy employed during multiple-step reaction trials (8% vs. 56.3%, *p *< 0.001). In addition, leg collision (Col) and unsuccessful recovery responses (i.e., a fall into the harness) resulted in all cases in the multiple-step trials.

## Discussion

In this study, our hypotheses were partially supported, as during the single-step trials, as the slip-like perturbation magnitudes increased, older people showed a small, yet significantly longer step initiation duration and step duration (R^2^ = 0.155 and 0.166, Fig. 1A, B). The spatial parameters, i.e., step length and step velocity, showed a larger increase than the temporal parameters of stepping as the perturbation magnitude increased (R^2^ = 0.272 and 0.254, respectively, Fig. 1C, D). This shows that the first recovery step, especially the spatial parameters, demonstrated a flexible behavior which we defined as the ability to adopt new kinematic movement patterns following changes in task requirements, i.e., the magnitude of perturbation [34]. This suggests that in the single-step trials, where the perturbation magnitudes were relatively low, an adaptive behavior was used, whereas longer and faster recovery steps were performed as the challenge of the test became greater, i.e., the perturbation magnitudes increased. This is supported by Pai and Patton [35] and Pai et al. [36], who demonstrated that the occurrence of a step depends on the interaction between the CoM position and its velocity. Following their model, stepping is necessary if there is a sufficiently high velocity of CoM displacement, even if the vertical projection of the CoM is located within the base of support (BoS) at step initiation.

The above results are in agreement with Vlutters et al. [25], who found that after exposing young participants to mediolateral pelvic pull perturbations at various magnitudes during treadmill walking, the step length, i.e., the foot placement after the perturbation, was adjusted proportionally to the mediolateral CoM velocity. McCrum et al. [36] revealed that a different mode of perturbation showed similar effects. They exposed their participants to repeated trip-like perturbations and found that after repeated perturbations of the left leg, older adults required fewer steps to recover their balance. Furthermore, Epro et al. [38] found that the neuromotor system in older adults shows rapid plasticity to repeated unexpected trip-like perturbations. Luchies et al. [39] suggested that the central nervous system estimates the level of instability following a balance perturbation, selects the appropriate balance recovery response, and pre-plans the stepping behavior, i.e., the use of one large step to recover balance or the use of several small steps, even before the first step is completed. This is supported by the results of Miyake et al. [39], who found that after exposure to repeated trip-like perturbations, the minimum toe clearance was modified toward more precise control and lower toe clearance of the swinging foot, which appears to reflect both the expectation of potential forthcoming perturbations and a quicker recovery response in cases of balance loss.

In the present study, we expand current knowledge by exploring balance reactive responses in cases where multiple steps were needed to recover balance, i.e., where the perturbation magnitudes were relatively high. Multiple-step responses were always performed at higher perturbation magnitudes than in the single-step trials; thus, they were more similar to a real-life balance loss threat. Interestingly, in the multiple-step trials, as the perturbation magnitudes increased, older people did not show changes in the timing of their first step initiation (Fig. 1A'), first step length (Fig. 1C'), or first step velocity (Fig. 1D'), and a small, yet significant increase was found for first step duration (Fig. 1B'). This suggests that at high perturbation magnitudes, i.e., during the multiple-step trials, when participants performed more than one step to recover their balance, their first recovery step performance exhibited a rigid behavior. We define this behavior as the inability to adopt new kinematic movement patterns following changes in task requirements, i.e., at increased perturbation magnitudes, suggesting a more automatic/stereotypical behavior during the first step of the multiple-step trials. However, the total balance recovery parameters, which represent the whole balance recovery, showed a significant increase (Fig. 2A-C), which suggests a flexible behavior. Thus, during the first recovery step of the multiple-step trials, older adults activate pre-programmed kinematic movement patterns of the extra steps, which are depended on the magnitude of perturbation, helping them to effectively recover balance trying to effectively "catch" the moving CoM over the BoS.

To fully understand the balance reactive response to increasing magnitudes, it must be noted that the kinematics of the first recovery step in the single- and multiple-step trials may be influenced by the strategy of the first recovery step. Our findings clearly show that during the single-step trials, as the perturbation magnitude increased, the LLSS was increasingly in use, from about 10% at low perturbation magnitudes to about 40% at the highest perturbation magnitudes (Fig. 2A). Meanwhile, the ULSS strategy decreased in use, from about 100% at the lowest perturbation magnitudes to about 10% at the highest magnitudes (Fig. 2B). With the LLSS strategy, there is a need to first unload the loaded leg, and then to swing the loaded leg sideways to perform the recovery step [2, 17]. It was reported earlier that the step initiation duration was delayed about 200 ms when an LLSS was performed compared to unloaded leg strategies [2, 17]. This, in fact, explains our findings that as the perturbation magnitude increased, the step initiation duration of the single-step trials also slightly increased (R^2^ = 0.155, Fig. 1A). Due to the unloading phase of the loaded leg in the LLSS, it took a longer time to initiate and complete the recovery step when the perturbation magnitude increased. This also resulted in a small, yet significant increase in the duration of step execution (R^2^ = 0.166, Fig. 1B). The different strategies that were used as the perturbation magnitudes increased further support the notion that the first stepping response in the single-step trials is flexible in nature and that participants selected a different strategy as the perturbation magnitudes increased (Fig. 2A). Since not only the timing of step initiation increased during the single-step trials, but also the strategies used changed as the magnitudes increased, it appears that older adults pre-plan their stepping performance, and perhaps a learning effect occurred during the experiment.

During the multiple-step trials, in which the older adults were exposed to higher perturbation magnitudes, as the magnitudes increased, they used similar first step strategies. The unloaded leg strategies, i.e., COS and ULSS, were the most commonly used, i.e., these made up about 70% of the strategies used across all perturbation magnitudes, while the LLSS strategy was rarely performed (16.7%) (Fig. 2B). This indicates that when older adults were exposed to large perturbation magnitudes, their strategies of balance responses also show rigid behavior. The COS strategy was performed because high perturbation magnitudes induce a faster CoM displacement to load the standing leg and unload the swing leg, allowing foot-lift of the unloaded leg to "crossover" the loaded leg. Since the initiation duration, step duration, and step length of these recovery strategies was somewhat similar across all perturbation magnitudes of the multiple-step trials (Fig. 1A'-D'), this supports the view that using the COS strategy was the best to control the moving CoM during the multiple-step trials at relatively high perturbation magnitudes, and that this response may not be under volitional control, thus, requiring an automatic response during the first recovery step. The present study results also indicate differences in balance recovery responses in walking [25, 27] versus standing. Both Vlutters et al. [25] and Nachmani et al. [27] found that as the perturbation magnitudes increased during self-selected treadmill walking, there were small yet significant decreases in the timing of the step response. In the present study, however, we found an increase in the timing of the step response as the magnitude increased, suggesting that there are differences in balance recovery responses in walking [25, 27] versus standing. We assume that these differences are related to specific learning effects to a different condition. One foot is usually more loaded during walking than the other leg, allowing the participants to easier react with the unloaded leg and learn how to perform the step even faster along with the experiment. In standing howver, both legs are equally loaded, and the delay in step initiation may be associated with learning how to better control, i.e., decelerate the moving CoM over the BoS.

Several limitations of this study should be acknowledged. First, the results are based on a sample of older people who had a relatively high function level, limiting generalization of these conclusions to frail older adults. Second, in the single-step trials, since the magnitude of unexpected perturbations always occurred in the same order (from low to high perturbation magnitudes), there may have been a learning effect that enabled some of participants to predict that the next perturbation would be higher and to "resist" stepping; thus, the duration of the first step initiation was somewhat delayed. This is supported by earlier studies that showed that when repeatedly applying perturbations, people show rapid plasticity and learning [25, 34–38, 40]. However, this would not have been the case in the multiple-step trials i.e., at relatively high perturbation magnitudes where the strategies and kinematics were similar across all perturbation magnitudes, and participants did not show different coordination patterns; thus, these results were not influenced by the perturbation order. Third, some may argue that the instructions we gave to the participants, i.e., "React naturally and try to avoid falling", may not have been appropriate, and that more constrained instructions such as "Try not to take a step" or "Step as rapidly as possible" would have been more appropriate. But we chose to use unconstrained instructions since these were likely to be more relevant for exploring the ability of the individual to respond in a real-life situation and are, thus, more ecologically valid. Fourth, out of 86 older adults, 18 asked to stop the experiment before completing all of the perturbation trials. Since these participants might have been "the weakest older adults" in our sample, this may have influenced the results in the high perturbation trials i.e., multiple-step trials as in Fig. 2, after magnitude 8, a small gradient decrease appears, especially in the step initiation duration as the magnitude increases.

## Conclusions

Results of the present study indicate that older people demonstrate a "flexible behavior" in their recovery step strategies and kinematics when a recovery step was initiated at relatively low perturbation magnitudes, i.e., single-step trials. However, at relatively high perturbation magnitudes, i.e., multiple-step trials, the kinematics and strategies of the first recovery step displayed a more "rigid behavior," suggesting a more automatic response, while the following steps, i.e., total balance recovery kinematics, were scaled to the perturbation magnitude, suggesting that older adults activated pre-planned programs based on the perturbation magnitude, right away after the unannounced perturbation, even before the.

## Supplementary Information

**Additional file 1: SupplementaryTable 1. **Characteristics of surface horizontal translation

## Data Availability

All data generated or analyzed during this study are included in this published article [and its supplementary information files] are not publicly available due to ethical considerations, but are available from the corresponding author on reasonable request.

## Abbreviations

CoM: Center of mass
MMSE: Mini Mental State Examination score
LLSS: Loaded leg side step
ULSS: Unloaded leg side step
COS: Cross-over step
Col: Leg collision
eCoM: Estimated CoM
ms: Milliseconds
cm: Centimeters
cm/sec: Centimeters per second
BMI: Body mass index
FES-I: Fall Efficacy Scale intenational
BoS: Base of support
