# Supplementary material for: Characteristics of step responses following varying magnitudes of unexpected lateral perturbations during standing among older people – a cross-sectional laboratory-based study
Source: BMC Geriatr. 2022 May 6;22:400. doi: 10.1186/s12877-022-03080-w (PMC9078012; doi:10.1186/s12877-022-03080-w)
Supplement: Supplementary file 1 — Additional file 1: SupplementaryTable 1. Characteristics of surface horizontal translation [file 12877_2022_3080_MOESM1_ESM.docx]

**Supplementary Table 1:** Characteristics of surface horizontal translation

| **Displacement** (cm) | **Displacement Time** (sec) | **Velocity** (cm/sec) | **Acceleration** (cm/sec^2^) |
| --- | --- | --- | --- |
| **1** | 0.30 | 6 | 25 |
| **2** | 0.40 | 7 | 60 |
| **3** | 0.45 | 11 | 80 |
| **4** | 0.50 | 14 | 85 |
| **5** | 0.55 | 15 | 90 |
| **6** | 0.60 | 17 | 94 |
| **7** | 0.65 | 19 | 98 |
| **8** | 0.68 | 21 | 102 |
| **9** | 0.70 | 22 | 107 |
| **10** | 0.73 | 23 | 111 |
| **11** | 0.75 | 25 | 117 |
| **12** | 0.78 | 26 | 123 |
| **13** | 0.80 | 28 | 128 |

The basic protocol on which all analyses were carried out included 13 perturbation magnitudes ranging from 1 to 13 for a total of 26 random right and left perturbation trials. Note: Data presented as peak Velocity and Acceleration.

cm = centimeters; sec = seconds; sec/cm = centimeters per second; sec/cm^2^ = centimeters per second squared.
